# Details and Updates From the Consensus Meeting on Anatomical Borders for ICG Usage in Urological Laparoscopic and Robotic Kidney Surgery

**DOI:** 10.1111/ases.70184

**Published:** 2025-11-13

**Authors:** Shintaro Narita, Junji Ichinose, Shinji Itoh, Satoshi Kobayashi, Shuichi Morizane, Daisuke Asano, Yujin Kudo, Toshiya Abe, Kenoki Ohuchida, Keiichi Akahoshi, Go Wakabayashi, Kimihiro Shimizu, Hisashi Iwata, Atsushi Takeneka, Minoru Tanabe, Masatoshi Eto, Norihiko Ikeda, Masafumi Nakamura, Yuko Kitagawa, Tomoharu Yoshizumi, Mingyon Mun, Tomonori Habuchi

**Affiliations:** ^1^ Department of Urology Akita University Graduate School of Medicine Akita Japan; ^2^ Department of Thoracic Surgical Oncology Cancer Institute Hospital of the Japanese Foundation for Cancer Research Tokyo Japan; ^3^ Department of Surgery and Science, Graduate School of Medical Sciences Kyushu University Fukuoka Japan; ^4^ Department of Urology, Graduate School of Medical Sciences Kyushu University Fukuoka Japan; ^5^ Division of Urology, Department of Surgery, Faculty of Medicine Tottori University Tottori Japan; ^6^ Department of Hepatobiliary and Pancreatic Surgery Institute of Science Tokyo Tokyo Japan; ^7^ Department of Surgery Tokyo Medical University Tokyo Japan; ^8^ Department of Surgery and Oncology, Graduate School of Medical Sciences Kyushu University Fukuoka Japan; ^9^ Department of Surgery Ageo Central General Hospital Ageo Japan; ^10^ Division of General Thoracic Surgery, Department of Surgery Shinshu University School of Medicine Matsumoto Japan; ^11^ Department of General Thoracic Surgery Gifu University Hospital Gifu Japan; ^12^ Department of Surgery Kashiwa Municipal Hospital Kashiwa Japan; ^13^ Department of Surgery Keio University School of Medicine Tokyo Japan

**Keywords:** fluorescent, ICG, kidney, renal cell carcinoma, robotic surgery

## Abstract

**Introduction:**

This study aimed to update the literature and present findings from a national survey on the current use and perceived utility of indocyanine green (ICG) fluorescence imaging in laparoscopic and robotic kidney surgeries, as discussed at the Consensus Meeting on Anatomy on the Border.

**Methods:**

This study consisted of two parts. First, a narrative review of previous studies on ICG application in kidney surgeries was conducted. Second, a questionnaire for urologists certified in laparoscopic surgery was created to evaluate current practices regarding ICG usage.

**Results:**

Nine studies on ICG use in partial nephrectomy were reviewed. The sole randomized controlled trial (RCT) found no significant benefit of ICG‐guided robotic‐assisted partial nephrectomy (RAPN), although the majority of observational studies suggested potential advantages. The lack of methodological standardization remains a major barrier to its wider implementation. Of the 114 urologists contacted, 32 (28%) responded, most of whom had over 20 years of surgical experience. Among the respondents, 31% reported using ICG in renal surgeries: 20% used it exclusively in RAPN, 60% in RAPN in combination with other procedures, and 20% in other surgeries. The dosing varied, but over half of the respondents used 12.5 mg per injection.

**Conclusion:**

Although observational data indicate the potential utility of ICG in renal surgery, the RCT finding is inconclusive, and its current use among urologists remains limited. Nevertheless, ICG holds promise for a broader application in urological procedures.

## 1 Introduction

At present, minimally invasive surgeries, including laparoscopic and robotic surgeries, are widely employed in the field of urology [1]. Introduced in 1947, fluorescence‐guided surgery revolutionized surgical techniques by providing real‐time visual feedback to support intraoperative decision‐making [2]. In particular, real‐time fluorescence navigation is well suited to minimally invasive procedures as fluorescence imaging can be seamlessly integrated into monitor‐based visualization systems. Moreover, the incorporation of fluorescence modes, such as Firefly, into robotic platforms, such as the da Vinci Si and Xi systems, has contributed to the widespread application of near‐infrared fluorescence (NIRF)‐guided surgeries.

Indocyanine green (ICG), a water‐soluble fluorophore, has been widely used in clinical research since its approval for intravenous administration by the US Food and Drug Administration in 1956 [2]. When stimulated by near‐infrared light, ICG emits green fluorescence that can be detected using specialized optical systems, without obstructing the surgical field [2]. ICG fluorescence imaging has emerged as a valuable tool across various surgical disciplines; it enables real‐time visualization of vascular structures and tissue perfusion. In urological surgery, the safety and versatility of ICG have contributed to its growing popularity [3, 4]. Previous studies have shown the feasibility of NIRF‐guided procedures using ICG in various urological surgeries, including kidney surgery, prostatectomy, cystectomy, and varicocelectomy [2, 5]. However, despite the expanding body of literature supporting its utility, the optimal indications for ICG use and real‐world practice patterns, particularly in Japan, remain unclear.

The Consensus Meeting on Anatomy on the Border, held by the Japanese Society of Endoscopic Surgery, proposed a multidisciplinary statement on the use of ICG across various organ systems, including the lung, liver, and kidney. This study aimed to update the narrative review of existing literature on ICG use in urological surgery and to present detailed findings from a questionnaire survey of urologic surgeons in Japan, which was conducted as part of the consensus meeting, with a particular focus on kidney surgeries.

## 2 Materials and Methods

### 2.1 Literature Review

An extensive search was performed on PubMed and medical journals for literature describing segment delineation and tumor localization using ICG fluorescence, focusing on English publications. The search terms included “indocyanine green,” “fluorescence,” “kidney,” and “surgery.” Randomized controlled trials (RCTs), meta‐analyses, prospective cohort studies, and large retrospective series were prioritized. Additional relevant studies were identified by reviewing the reference lists of the selected articles. Further eligible publications were obtained through manual cross‐referencing of the bibliographies of previously included studies.

### 2.2 Survey Design and Distribution

A web‐based questionnaire was created and emailed to 114 urological laparoscopic surgeons certified by the Japan Society for Endoscopic Surgery (JSES). The questionnaire covered the following topics: (1) surgical experience and subspecialty focus; (2) indications for ICG use; (3) ICG administration protocols, including dosage and timing; (4) perceived ICG benefits and limitations; and (5) procedures for fluorescence imaging detection. The survey was conducted anonymously from August to September 2024. The summarized statement of the Consensus Meeting on Anatomical Borders based on the results of this questionnaire survey is reported separately.

## 3 Results

### 3.1 Literature Review of ICG Usage in Partial Nephrectomy

Since its introduction approximately 15 years ago, ICG has been widely used in nephron‐sparing surgeries [2]. During endoscopic partial nephrectomy, ICG is mainly used for two purposes: evaluating renal perfusion following arterial clamping and distinguishing between normal parenchyma and renal cell carcinoma based on differential fluorescence patterns, thereby facilitating tumor resection [5]. Incomplete ischemia can lead to excessive bleeding, which may impair surgical visualization, resulting in incomplete tumor removal and potential complications. Furthermore, some studies have explored the differential binding affinity of ICG between kidney tumors and healthy parenchyma, with tumors often exhibiting hypofluorescence. This contrast potentially facilitates tumor identification and improves resection accuracy [6].

In 2011, Tobis et al. reported the initial clinical application of NIRF with ICG in 11 patients undergoing robotic‐assisted partial nephrectomy (RAPN). They aimed to differentiate normal from malignant tissues and to visualize the renal vasculature [7]. However, Manny et al. concluded that ICG‐based classification did not reliably distinguish benign from malignant lesions [8]. ICG‐NIRF‐guided selective arterial clamping is known to provide surgeons with an intraoperative renal angiogram, allowing for selective clamping of segmental arteries rather than the main renal artery. Several studies have explored the use of ICG to preserve renal function and ensure negative surgical margins during PN (Table 1). Given that eight studies [9, 10, 11, 12, 13, 14, 15, 16] were reviewed in the Consensus Meeting on Anatomical Borders and one comparative study [6] was recently published, we re‐reviewed nine studies on RAPN or laparoscopic partial nephrectomy, including one RCT, one prospective study, and seven retrospective comparative studies (Table 1).

**TABLE 1:** Previous literature to investigate the impact of ICG‐guided robot‐assisted partial nephrectomy.

Authors	Year	Design	Induction method	ICG dosage	Intervention versus control	Number of patients	Summary of results
Borofsky	2012	Retrospecitve	NA	NA	Zero ischemia RAPN versus c‐RAPN	27 versus 27	Longer operating time and superior kidney function (~2w)
Harke	2014	Retrospective	IV	~5 mg	Selective versus Global ischemia	15 versus 15	Selective clamping associated with superior kidney function at discharge
McClintock	2014	Retrospecitve	IV	5–7.5 mg	ICG with selective clamping	42 versus 42	Selective clamping tended to be associated with superior kidney function at 3 m
Mattevi	2019	Retrospective	IV	5 mg	ICG‐RAPN versus c‐RAPN	15 versus 42	A greater loss of GFR at 1 m in c‐RAPN
Diana	2020	Retrospective	IV	5–10 mg	All ICG + RAPN	318	No difference in complication and trifecta in selective or conventional
Sentell	2020	Prospective	IV	0.625–1.25 mg	All ICG‐RAPN	361	Good surgical margin positivity (0.3%)
Long	2022	RCT	IV	NA	Super selective‐RAPN versus c‐RAPN	15 versus 14	No difference in %eGFR (6 m)
Yang	2022	Retrospective	IV	NA	ICG‐RAPN versus c‐RAPN	21 versus 106	Longer operative time and superior eGFR (3 m)
Joffe	2025	Retrospective	IV	7 mL	ICG‐RAPN/LPN versus c‐RAPN	87 versus 63	ICG use associated with change in CKD stage (OR 9.9, 95% CI: 1.0–93.9, *p* = 0.05).

Abbreviations: c‐RAPN, conventional robot‐assisted partial nephrectomy; GFR, glomerular filtration rate; LPN, laparoscopic partial nephrectomy; NA, not assessed; RAPN, robot‐assisted partial nephrectomy; RCT, randomized clinical trial.

In 2012, Borofsky et al. conducted a matched‐pair analysis, comparing the surgical outcomes between ICG‐guided selective arterial clamping and conventional main renal artery clamping, all performed by a single surgeon [9]. They reported that zero‐ischemia RAPN with superselective clamping performed in 79.4% of 34 patients was safe and feasible. They also reported a longer operative time but better postoperative kidney function (~2 w). Several retrospective studies have since reported that ICG use for selective clamping results in enhanced preservation of postoperative glomerular filtration rate; however, they lack data on intermediate‐ and long‐term renal outcomes [10, 11, 12]. In 2022, Yang et al. also observed a short‐term benefit of eGFR preservation in ICG‐guided RAPN [16], with longer operative times but enhanced renal function at 3 months postoperatively. Long et al. conducted the sole RCT on this topic—the EMERALD trial—comparing superselective RAPN with ICG versus conventional RAPN using the da Vinci Si system [15]. The trial adopted a transperitoneal approach but was prematurely terminated for futility owing to the absence of a favorable trend in surgical or functional outcomes. Despite the relatively small sample size, no significant differences were observed in the postoperative eGFR between the groups, as presented in Table 1. Furthermore, in a multicenter retrospective cohort study involving 737 patients, Diana et al. reported that ICG‐guided RAPN was associated with improved trifecta outcomes and no major complications (Clavien–Dindo grade > 2) [13]. The study evaluated surgical success using two criteria: the MIC (minor ischemic complications) criteria, defined as the absence of Clavien–Dindo > 2 complications, WIT < 20 min, and negative surgical margins, and the trifecta criteria, defined similarly, but with WIT < 25 min. This multicenter study included patients who underwent RAPN between 2010 and 2016. Among them, 318 had complete demographic and clinical data and underwent ICG‐guided RAPN.

Although the MIC achievement rate did not considerably differ between ICG‐guided and conventional RAPN, the former achieved a higher trifecta success rate of approximately 80%. However, a key limitation of this study was the absence of a direct comparison with a non‐ICG group.

In a more recent single‐center retrospective study, Joffe et al. examined the outcomes of ICG use in 150 RAPN cases [6]. ICG was used in 58% of the cases, but no significant advantages were observed in terms of ischemia time, estimated blood loss (EBL), or the rate of positive surgical margins. Notably, ICG use was associated with increased risk of postoperative chronic kidney disease stage progression (odds ratio, 9.9; 95% confidence interval, 1.0–93.9; *p* = 0.05).

A recent meta‐analysis involving six retrospective studies—including several of those reviewed above—revealed a statistically significant reduction in ischemia time in the ICG group (weighted mean difference: 1.46 min) but no differences in EBL or positive surgical margin rates. In addition, a slight improvement in eGFR was observed in the ICG group at 1–3 months postoperatively (weighted mean difference: +9.26 mL/min), whereas no significant difference was observed in the percentage decline in eGFR at discharge. The ICG group also had a shorter ischemia time (weighted mean difference: −1.46 min), but there were no differences in EBL and positive surgical margin rates. Moreover, improvements in eGFR at 1–3 months postoperatively (weighted mean difference: +9.26 mL/min) were observed, whereas there was no difference in %eGFR decline at discharge [17].

In summary, eight of the nine studies compared the outcomes between ICG‐guided RAPN and conventional RAPN [6, 9, 10, 11, 12, 13, 14, 15, 16]. Among these studies, five reported that ICG‐guided RAPN improved postoperative renal function, reduced hospital stay, and decreased operative time [9, 10, 11, 12, 16]. One prospective, single‐center study with a relatively large patient cohort found that ICG‐guided RAPN was associated with a low positive surgical margin rate [14]. A recent higher number study also reported that ICG‐guided RAPN did not show significant superiority to conventional RAPN [6]. ICG is commonly used in RAPN to visualize segmental renal arteries and delineate the perfusion boundaries. Although numerous studies have reported the benefits of ICG‐guided RAPN over conventional RAPN, the RCT and two relatively large studies did not yield positive results, and the meta‐analysis did not review very recent negative studies highlighting the need for further investigation.

Cortical tumors have been demonstrated to downregulate bilitranslocase, a carrier protein responsible for intracellular ICG uptake and typically absent in most cancerous cells [2, 18]. The characteristics result in differential fluorescence between the tumors and the surrounding normal parenchyma, allowing for potential intraoperative visual demarcation.

Sentell et al. investigated tumor demarcation using ICG by exploring tumor detection during RAPN, conducted between June 2011 and March 2018, using a prospectively collected database of 361 consecutive cases [14]. The patients received a relatively low dose of ICG (0.625–1.25 mg IV), and 288 tumors (87.3%) exhibited differential fluorescence. In the predominant histologies, 249 of 277 (89.9%) renal cell carcinomas did not fluoresce, whereas 23 of 32 (71.9%) oncocytomas did not. In this cohort, the positive surgical margin rate was notably low at 0.30%. Although the findings suggest the potential for fluorescence‐guided navigation in RAPN, the study was conducted by a single surgeon and lacked a control group for comparison of margin outcomes. Therefore, further controlled, multi‐institutional studies are warranted to confirm the efficacy and generalizability of this technique.

### 3.2 Current Landscape and Other Applications of ICG for Kidney Surgery on Literature

Simone et al. proposed an innovative use of ICG for endophytic renal masses by introducing a preoperative superselective transarterial delivery of an ICG–Lipiodol mixture into tertiary‐order arterial branches supplying the tumor prior to transperitoneal off‐clamp RAPN [19]. The authors emphasized several potential benefits, including enhanced preoperative planning, rapid intraoperative tumor localization, and real‐time visualization of resection margins. The feasibility of this “tattooing” technique has since been supported by several subsequent studies [20, 21]. In 2022, Nardis et al. examined the clinical impact of this approach in a cohort of 41 patients with endophytic renal tumors [22]. They reported a procedural success rate of 100%, with 63.4% of tumors considered to be “visible with well‐defined margins” during surgery. In addition, Amparore et al. reported the utility of overlaying ICG imaging onto a 3D virtual model of the kidney aligned with the actual organ during surgery, further enhancing intraoperative guidance and anatomical orientation [23].

ICG has also been used in other areas of renal surgery. Aslim et al. reported its potential utility in renal transplantation involving complex vascular cases (PMID: 30210867). Boni et al. reported the use of ICG during laparoscopic procedures in 108 patients, including 8 who underwent donor nephrectomy and 1 who underwent autologous renal transplantation (PMID: 25303914). However, the clinical utility of ICG during kidney transplantation and donor nephrectomy remains to be clearly established.

## 4 Survey Findings

**FIGURE 1:**
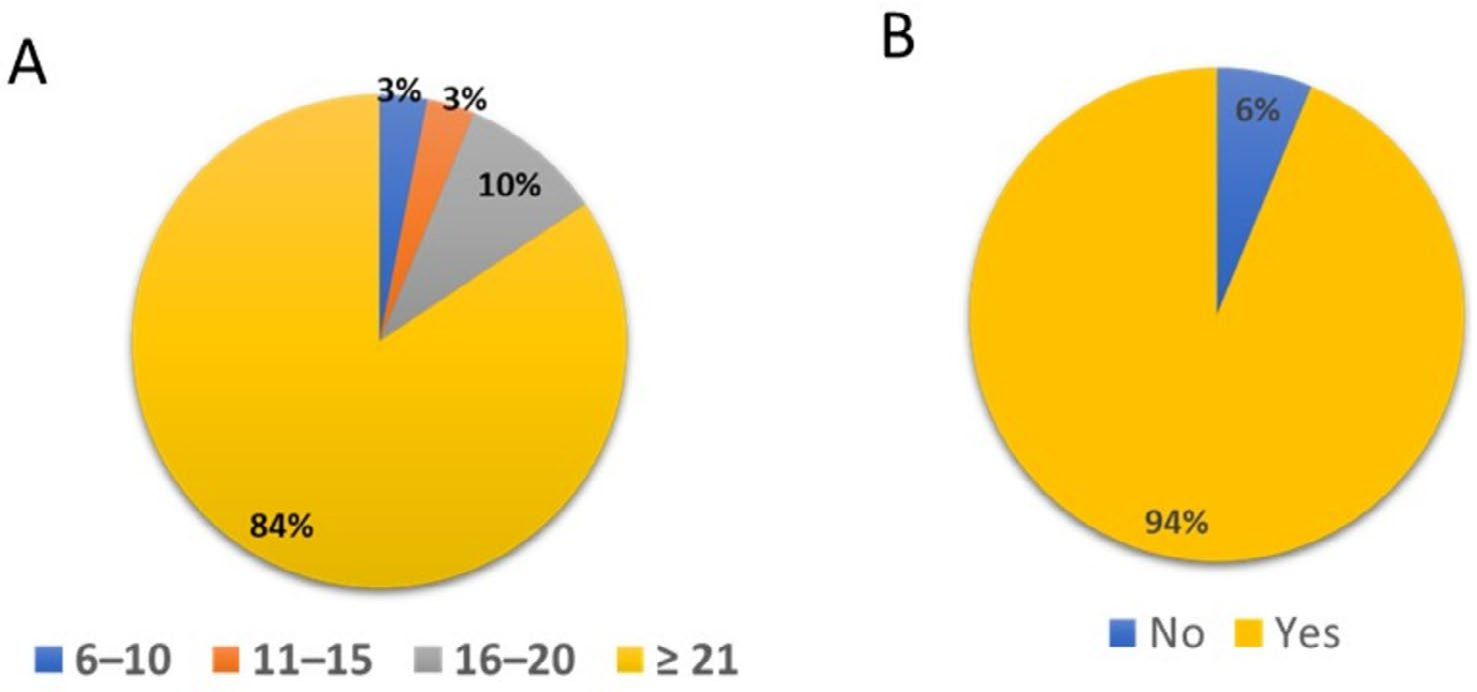
Results of the questionnaire survey on the backgrounds of the surveyed urologists. (A) How many years of experience do you have as a surgeon? (B) Do you have certification in laparoscopic urological surgery?

**FIGURE 2:**
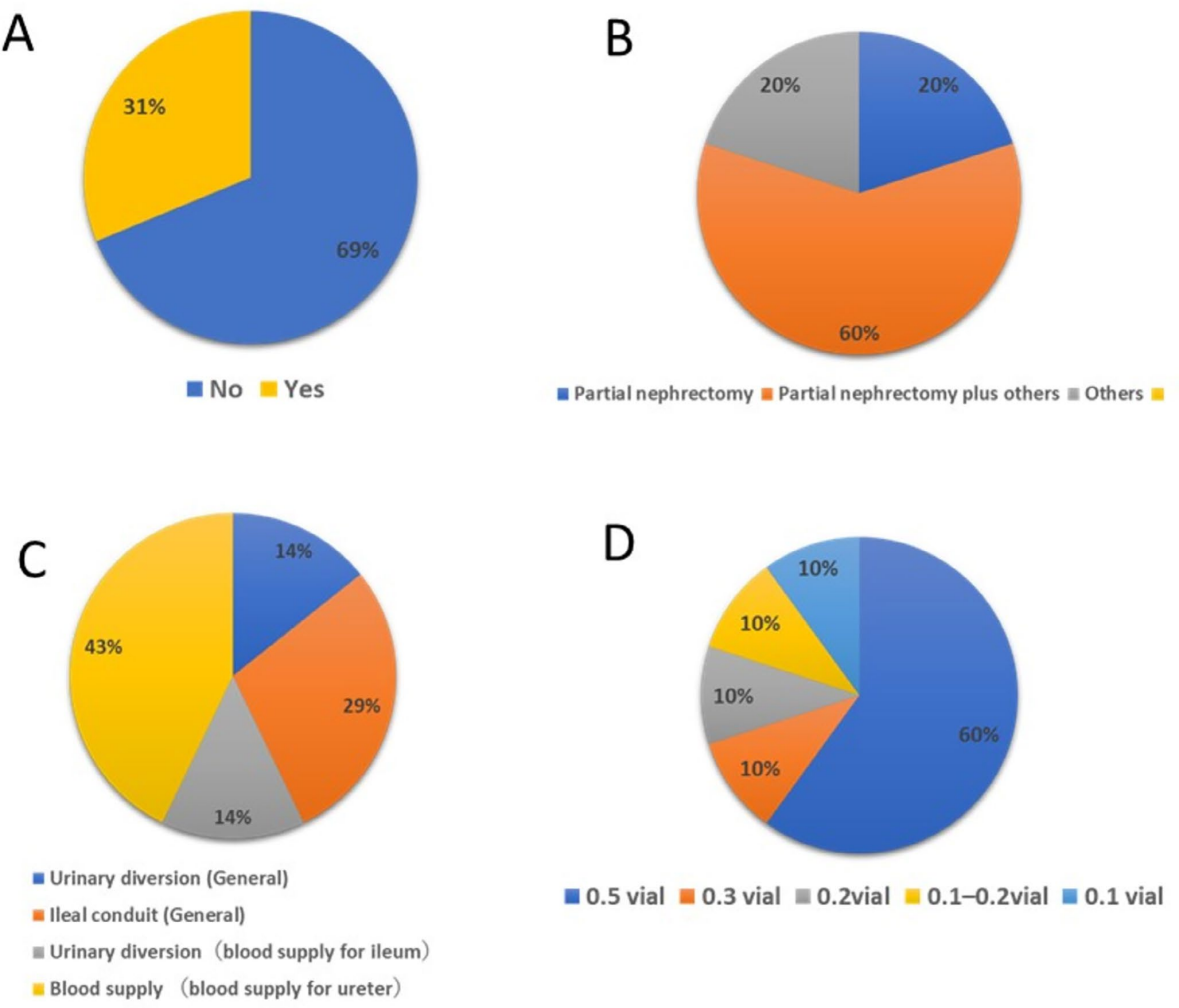
Results of the ICG‐related questionnaire. (A) Do you routinely use ICG to identify regions within the kidney? (B) For which procedures do you think ICG provides significant benefits? (C) For which types of surgery, other than kidney surgery, do you use ICG? (D) What is the dosage of ICG you use to identify regions within the kidney in your practice?

Next, we showed the details of a survey regarding the current situation of ICG use by urology experts for endoscopic surgeries. The study included 114 urologists who were JSES‐certified laparoscopic surgeons (Figures 1 and 2). A total of 32 individuals responded to the survey, yielding a 28% response rate (Figure 1). Among the respondents, 84% had more than 21 years of urology experience (Figure 1A), whereas only 3% had less than 10 years. In addition, 94% of the participants were certified in laparoscopic urological surgery by the Japanese Society of Endoscopic and Robotic Surgery (Figure 1B).

Only 31% of the respondents used ICG in their renal surgeries (Figure 2A). Among them, 20% used ICG exclusively in RAPN, 60% in RAPN in combination with other procedures, and 20% in other types of renal surgeries (Figure 2B). The use of ICG in nonrenal urologic procedures was also investigated. Among the respondents, 57% used ICG in urinary diversion and 43% used it to assess ureteral blood supply (Figure 2C). In summary, despite the relatively low overall frequency of ICG use in kidney surgeries among Japanese urologists, ICG was used across a range of urologic procedures.

A considerable variation was observed in the dosage of ICG used. However, the majority of respondents (60%) reported using 0.5 vial (equivalent to 12.5 mg) per administration. The other reported doses were 0.1, 1.0–2.0, 2.0, and 3.0 vials, each accounting for 10% of the responses (Figure 2D). These results suggest that Japanese urologists tend to use relatively higher doses of ICG compared with those reported in previous studies.

## 5 Discussion

In the first part of this study, the literature review regarding the use of ICG in laparoscopic and robotic kidney surgeries was updated for the consensus meeting of the Anatomy of Border Committee within the Japanese Society of Endoscopic Surgery. Most studies have focused on partial nephrectomy, indicating that ICG has potential utility in this procedure. Although an RCT and two relatively large studies did not clearly demonstrate the benefits of ICG use, several observational studies, including a meta‐analysis, reported enhanced postoperative renal function with ICG‐guided RAPN. In addition, detailed results from a questionnaire survey conducted among Japanese experts in laparoscopic and robotic surgery during the consensus meeting were presented. The survey concluded that consensus on the routine use of ICG during kidney surgeries is currently lacking. Based on our narrative review and the survey results, ICG use has not yet been standardized, although it is applied in various minimally invasive urologic procedures.

ICG is a fluorescent dye developed by Kodak Research Laboratories in 1955 [24]. When intravenously injected, it rapidly binds to plasma proteins, confining it to the intravascular compartment [24]. The vasculature becomes fluorescent within less than a minute, with surrounding tissues becoming fluorescent within seconds thereafter. ICG has a plasma half‐life of 150–180 s and is exclusively excreted into the bile by the liver, enabling repeat injections approximately every 15 min during surgery [24]. Notably, ICG is non‐nephrotoxic and is solely eliminated via hepatic clearance. Furthermore, minor and major adverse reactions rarely occur [25], making it suitable for use in urologic renal surgery.

The results of the questionnaire survey indicated that Japanese urologists do not routinely use ICG during RAPN. As presented in Table 1, the lack of strong evidence may influence their decision‐making, despite several studies demonstrating the benefits of selective clamping with ICG during RAPN. Moreover, previous research has shown that long‐term renal function is more closely correlated with renal volume preservation than with ischemia time [11], which may contribute to surgeons' skepticism regarding the impact of selective clamping in RAPN.

As regards the ICG dosage, the recommended safe range for standard diagnostic procedures is 0.1–0.5 mg/kg [26]. Ferroni et al. proposed that 2.5 mg is an appropriate standard dose to administer immediately after arterial clamping during surgery [5]. However, the rationale for determining the optimal induction dose remains unclear. Further studies are warranted to elucidate the optimal ICG dosage for urologic surgeries, including kidney procedures, particularly in Japanese patients.

Several previous reviews have proposed the potential advantages of ICG use in urological surgeries beyond kidney‐related procedures [2, 5, 24]. One area of particular interest is its role in guiding the use of lymph node dissection templates in prostate and bladder cancer surgery [2]. When ICG is directly injected into the tissues, it migrates through the lymphatic vessels to the lymph nodes, where it accumulates in the macrophages, thereby providing valuable information on an organ's lymphatic drainage [2]. Several potential applications of ICG have also been reported during the reconstructive phase of cystectomy, for example, mesenteric angiography and ureteral vascularization assessment [2]. Consistent with this, our questionnaire survey found that many urologists used ICG during the reconstructive phase. Ahmadi et al. demonstrated a statistically significant reduction in the per‐patient rate of anastomotic stricture in the ICG group compared to the non‐ICG group (0% vs. 10.6%, *p* = 0.020) [27]. We previously reported that ICG‐guided perfusion assessment reduced the incidence of *de novo* hydronephrosis in robot‐assisted cystectomy [28]. Finally, robotic reconstructive procedures, such as ureterolysis, pyeloplasty, and ureteroureterostomy, may benefit from the use of ICG to locate strictures and confirm adequate ureteral perfusion [24]. The mechanism underlying the intraureteral use of ICG remains unclear [24], although one hypothesis suggests that ICG binds to urothelial surface proteins in viable ureteral tissue [29]. Further studies are warranted to investigate additional targets and elucidate the underlying mechanisms of ICG action in these contexts.

Several limitations of ICG use have also been reported. For instance, Backer highlighted some weaknesses of ICG instillation [30]. First, ICG provides information only on surface perfusion and does not enable visualization of the three‐dimensional tumor resection bed. Second, effective ICG imaging requires complete removal of surrounding fat from the kidney, which can be challenging in some cases. Although allergic reactions to ICG are rare, they have been reported in the context of RAPN [31].

## 6 Conclusion

Previous studies have demonstrated the utility of ICG in renal surgery, including RAPN; however, the RCT has reported no significant difference, and matched cohort studies are limited by short‐term follow‐up. At present, only a limited number of urological surgeons use ICG to delineate regions within the kidney, indicating that its use is not yet widespread among urologists performing kidney surgery. Although ICG has several potential applications in urologic surgery, further research is warranted to elucidate the strengths and limitations of different ICG techniques in minimally invasive procedures. Moreover, well‐designed comparative studies are necessary to determine the true impact of ICG‐guided urologic surgeries.

## Ethics Statement

The authors have nothing to report.

## Conflicts of Interest

Kenoki Ohuchida, Atsushi Takenaka, Tomonori Habuchi and Mingyon Mun are the Editorial Board members of ASES Journal and the co‐authors of this article. To minimize bias, they were excluded from all editorial decision‐making related to the acceptance of this article for publication. Shintaro Narita, Junji Ichinose, Shinji Itoh, Satoshi Kobayashi, Shuichi Morizane, Daisuke Asano, Yujin Kudo, Toshiya Abe, Kenoki Ohuchida, Keiichi Akahoshi, Kimihiro Shimizu, Hisashi Iwata, Atsushi Takeneka, Minoru Tanabe, Masatoshi Eto, Norihiko Ikeda, Tomoharu Yoshizumi, Mingyon Mun, and Tomonori Habuchi have no conflicts of interest to disclose. Go Wakabayashi has received honoraria from Stryker Japan K.K., Olympus Corporation, Johnson & Johnson K.K., Intuitive Surgical G.K., Covidien Japan Inc., and Sysmex Corporation, Medicaroid Corporation, and research funding from Medicaroid Corporation, Anaut Inc., and AMIN K.K. Masafumi Nakamura has received research funding from Olympus Corporation, Taiho Pharmaceutical Co. Ltd., Covidien Japan Inc., Chugai Pharmaceutical Co. Ltd., Eli Lilly Japan K.K., and Otsuka Pharmaceutical Co. Ltd., and honoraria from Intuitive Surgical G.K., Johnson & Johnson K.K., Yakult Honsha Co. Ltd., Taiho Pharmaceutical Co. Ltd., Daiichi Sankyo Co. Ltd., Otsuka Pharmaceutical Co. Ltd., Novartis Pharma K.K., Olympus Corporation, Covidien Japan Inc., and Servier Japan Co. Ltd. Yuko Kitagawa has received honoraria from Sysmex Corporation, Medicaroid Corporation, Olympus Corporation, Stryker Japan K.K., Intuitive Surgical G.K., and Ethicon Inc., and research funding from Medicaroid Corporation.

## Data Availability

Derived data supporting the findings of this study are available from the corresponding author S.N. on request.
